# 

**Published:** 1887-09-17

**Authors:** 


### THE HOSPITAL

An Institution, Family, and Parochial Journal of Hospitals, Asylums, & all Agencies for the Care of the Sick, Criticism & News.

### CONTENTS.

**"The Destruction of the Poor"** - - - 407

**Words of Consolation.**—Chapter L. The Sufferings of Christ - - - - - - - 408

**The Doctor's Pulpit.** – King Solomon's Physic - 409

**Till the Doctor Comes.** — XXVI. About Scarlet Fever - - - - - - - 410

**Nursing Institutions, Nurses, and the Women's Jubilee Offering.** By HENRY C. BURDETT - - - - - - - 411

**A Woman's Sacrifice.—For Duty's Sake** - 412

**Hospital Administration. — Alcohol and Milk Consumption** - - - - 413

**Notes and News.** — Miscellaneous Items. — Dogs as Collectors.— Guessing Competitions.— Hospital Fêtes and Demonstrations. — Horncastle and the Lincoln County Hospital.—The Brighton Dental Hospital.—Vacancies. — Volunteer Collectors. — The Folkestone Hospital Dispute.—The Royal Cornwall Infirmary.—The Pudsey Football Club.—The Workmen's Industrial Exhibition.—The Glasgow Medical Charities, etc., etc. - 414

**Annotations.**—Medical Students.—Medical Teachers. —Medical Examiners - - - - - 417

**Kate Mountstephen.** By C. G. FURLEY.—Chap. IV. Sister Katherine - - - - - - 418

**Where to Buy "The Hospital"** - - - 420

**"A Matron's Corner"** - - - - - 420

**Questions and Answers** - - - - 420

**The Children's Corner.**—Puzzles, Answers, Letters, etc. - - - - - - - - 421

**Amusements with Profit** - - - - 421

**In- and Out-Patients' Hospital Diary.**— General and Special Hospitals - - - - 422

